# An AI-Based Algorithm for the Automatic Classification of Thoracic Radiographs in Cats

**DOI:** 10.3389/fvets.2021.731936

**Published:** 2021-10-15

**Authors:** Tommaso Banzato, Marek Wodzinski, Federico Tauceri, Chiara Donà, Filippo Scavazza, Henning Müller, Alessandro Zotti

**Affiliations:** ^1^Department of Animal Medicine, Production and Health, University of Padua, Legnaro, Italy; ^2^Department of Measurement and Electronics, AGH University of Science and Technology, Krakow, Poland; ^3^Information Systems Institute, University of Applied Sciences - Western Switzerland (HES-SO Valais), Sierre, Switzerland

**Keywords:** cat, thorax, artificial intelligence, convolutional neural network, radiology

## Abstract

An artificial intelligence (AI)-based computer-aided detection (CAD) algorithm to detect some of the most common radiographic findings in the feline thorax was developed and tested. The database used for training comprised radiographs acquired at two different institutions. Only correctly exposed and positioned radiographs were included in the database used for training. The presence of several radiographic findings was recorded. Consequenly, the radiographic findings included for training were: no findings, bronchial pattern, pleural effusion, mass, alveolar pattern, pneumothorax, cardiomegaly. Multi-label convolutional neural networks (CNNs) were used to develop the CAD algorithm, and the performance of two different CNN architectures, ResNet 50 and Inception V3, was compared. Both architectures had an area under the receiver operating characteristic curve (AUC) above 0.9 for alveolar pattern, bronchial pattern and pleural effusion, an AUC above 0.8 for no findings and pneumothorax, and an AUC above 0.7 for cardiomegaly. The AUC for mass was low (above 0.5) for both architectures. No significant differences were evident in the diagnostic accuracy of either architecture.

## Introduction

Plain radiographs are, nowadays, a widely used diagnostic imaging tool used in the veterinary clinical routine to investigate the thorax in small animals. Despite the increasing availability of more advanced imaging techniques, such as computed tomography, plain radiographs are, in most cases, the first screening technique for thoracic disease. Furthermore, often the decision whether to perform additional, and more advanced, imaging investigations is based on the results of plain radiographs. In such a scenario, the correct interpretation of plain radiographs is paramount in prescribing successful treatment. However, the reported incidence of interpretation errors (in human medicine) for trained radiologists is still around 10–15% (1–3). The incidence of interpretation errors in veterinary medicine has not yet been reported.

Several strategies to reduce the incidence of interpretation errors have been proposed. While some non-technological solutions, such as structured reports, reductions in multitasking, and double readings, alone or combined, have been reported as decreasing inattention-related errors, on the other hand technological solutions such as eye-tracking technologies or computer-aided detection (CAD) software have also been proposed (3). The increasing availability of computers with a high computing power has driven the current research trend in the direction of the development of artificial intelligence (AI)-based CADs (4, 5). In fact, AI application in radiology is a major field of research with massive ongoing investments (6, 7). Currently, in human medicine, the main applications of AI on plain radiographs are related to the automatic detection of findings or pathologies (7, 8). In the veterinary field, the scope to use AI-based algorithms to detect some radiographic findings has been explored in dogs over the last few years (9–12). The same potential use has not yet been explored in cats.

Therefore, the aims of this study were: (1) to develop an AI-based CAD algorithm to automatically detect some of the most common radiographic findings in cats (2) to compare the diagnostic accuracy of some of the most commonly used convolutional neural network (CNN) architectures on our database.

## Materials and Methods

### Database Creation

All the feline radiographs performed at the Veterinary Teaching Hospital of the University of Padua between June 2010 and March 2021 and at the Pedrani Veterinary Clinic between December 2018 and November 2019 were included in the database. Three different X-ray equipments were used: at the Veterinary Teaching Hospital of the University of Padua a Kodak Point of Care CR-360 System (Carestream Health Inc.) was used from June 2010 to June 2018, whereas a FDR D-EVO 1200 G43 (Fujifilm Corporation) digital radiology (DR) is currently in use. At the Pedrani Veterinary Clinic a Isomedic RT 800 MA (Isomedic S. r. L) X-ray equipment was available. The PACS were interrogated to search for thoracic radiographs.

### Radiographic Findings

All the images were individually evaluated by two of the authors, TB and AZ with over 10 and 20 years' experience in small animal diagnostic imaging, respectively. All the radiographs were evaluated simultaneously by the two authors and the interpretation was concorded following a consensus discussion. Only correctly positioned and exposed radiographs were included in the database. Furthermore, only radiographs of skeletally mature cats were used. The radiographic findings were annotated, in a standardized fashion, for each radiograph. In particular, the presence of the following was recorded: alveolar pattern, bronchial pattern, interstitial pattern, cardiomegaly, pleural effusion, pneumothorax, fracture, hernia, megaoesophagus, pneumomediastinum, and subcutaneous emphysema (pneumoderma). If a radiograph was within normal limits, a “no findings” tag was applied. Bronchial or interstitial pattern were recorded as either present or absent, and no grading score was used. Cardiomegaly was defined based on the recommendations reported in the literature (13); in particular, all cats with abnormalities in both the size and shape of the cardiac silhouette (e.g., bulging of the right or left atrium) were classified as having cardiomegaly. The cardiac silhouette was evaluated, when possible, on both lateral and ventrodorsal and dorsoventral projections. If cardiomegaly was detected in one of the available projections, all the radiographs of the same animal were classified as cardiomegaly even if cardiomegaly was not evident in all the available projections. Both diffuse and segmental megaoesophagus were classified as megaoesophagus. The site of fractures was not recorded. Radiographs showing fractures of the hind limbs were discarded.

### Image Analysis

The images were stored in the lossless MHA format before being fed to the data loader. The processing pipeline started with resizing of the images to a 224 × 224 pixel format; these were then normalized to a (0–1) range. Classification was performed using a convolutional neural network (CNN), which is a group of deep-learning architectures specifically for image classification, segmentation and registration. Two different CNN architectures were evaluated, namely ResNet-50 (14) and Inception V3 (IncV3) (15). The CNN weights were initialized by pre-training the network using the ImageNet database and then they were fine-tuned on the database. A multi-label approach was opted for because different radiographic findings are usually present simultaneously. Binary cross-entropy was used as a cost function. The training hyperparameters were shared by the networks and this process was performed using the Adam optimizer together with an exponentially decaying learning rate until convergence was reached. The model state showing the epoch with the lowest loss in the validation set was chosen for further testing. The training cases were augmented by random cropping, affine warping, flips, and contrast changes. These augmentations apply random transformations to increase the dataset diversity. This is a standard, and commonly used, technique to improve the generalizability of deep networks by reducing the risk of over fitting the training set. The images were randomly splitted into a training, validation, and test set with a 8:1:1 ratio, respectively; an algorithm maintaining the same ratio among different tags in training, validation, and test set was used. The information regarding the institution was not used for training. The performance of the trained model on the test set is reported. No cross-validation was used. A purpose-built deep-learning workstation equipped with four graphics processing units was utilized for training (4× Tesla V100; Ubuntu 18.04, NVIDIA and Canonical). The evaluation metrics were not directly optimized during training.

### Statistical Analysis

All the statistical analyses were performed using a custom-built Python programming language script (Python Software Foundation; the Python Language Reference, version 3.6; available at http://www.python.org). The performance of the two architectures on individual radiographic findings was assessed with the area under the receiver operating characteristic curve (AUC). The performances of the two architectures were compared with the DeLong test. The differences in the AUCs of the considered tests, as a result of the DeLong test, are expressed as a *Z*-score. All *P-*values were assessed at an alpha of 0.05. The overall accuracy within the test set for both CNN architectures was also calculated.

## Results

### Database

One thousand six hundred and thirty-seven latero-lateral (LL) radiographs and 1,105 ventro-dorsal (VD) radiographs were retrieved. 575 LL radiographs and 426 VD radiographs were discarded due to poor positioning or incorrect exposure. Consequently, the database was composed of 1,062 LL and 679 VD radiographs. Due to the limited number of available VD radiographs, the CNN was trained only on the LL radiographs. The number of radiographs showing each radiographic finding is reported in Table 1.

**Table 1:** Summary of the radiographic findings for the LL radiographs.

**Radiographic finding**	**Number of radiographs**
No finding	571
Cardiomegaly	186
Bronchial pattern	120
Pleural effusion	115
Alveolar pattern	79
Mass	54
Pneumothorax	50
Interstitial pattern	29
Megaesophagus	17
Hernia	10
Fracture	3
Pneumomediastinum	3

### Selection of Radiographic Findings

**Figure 1:**
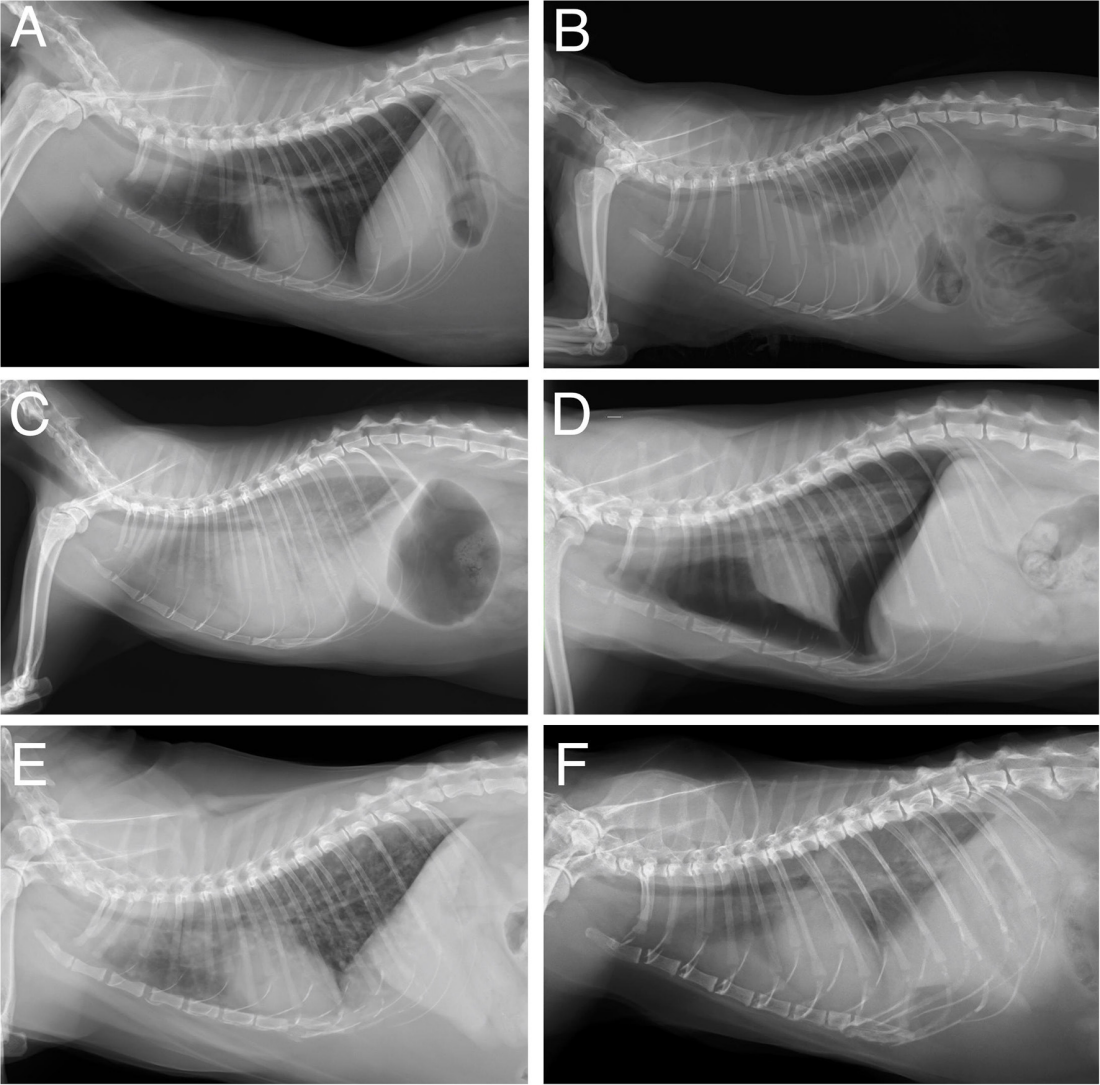
Example images of the radiographs used to train the CNN. **(A)** No finding; **(B)** pleural effusion; **(C)** cardiomegaly, alveolar, and interstitial pattern; **(D)** pneumothorax and alveolar pattern; **(E)** bronchial pattern; **(F)** mass, interstitial pattern, and alveolar pattern.

Some of the included radiographic findings (fracture, hernia, megaoesophagus, interstitial pattern, pneumomediastinum, and pneumoderma) were scarcely represented in the database and, therefore, were not included in training. Consequently, the findings included for training were: no findings, bronchial pattern, pleural effusion, mass, alveolar pattern, pneumothorax, cardiomegaly (Figure 1).

### Classification Results

**Figure 2:**
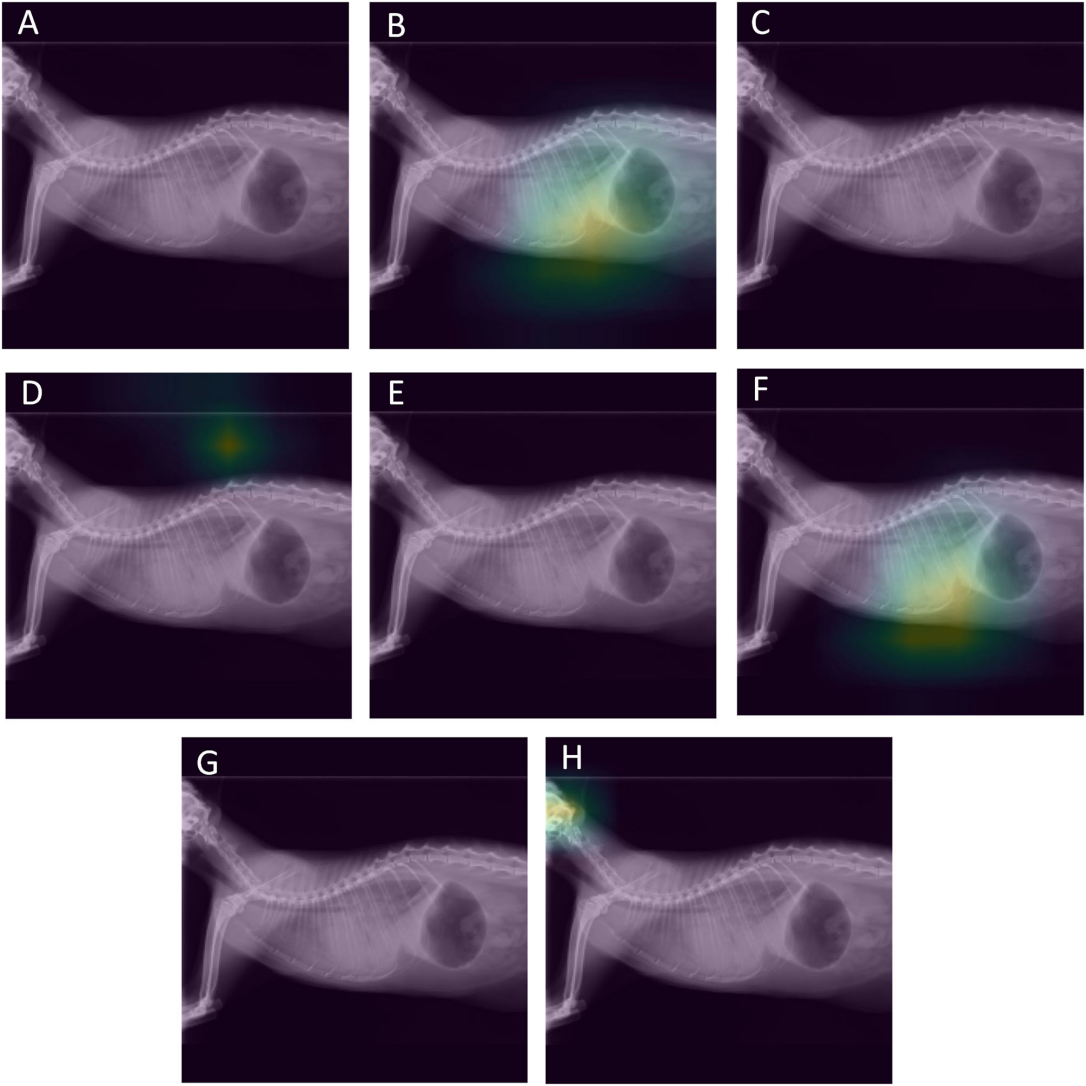
Visual assessment of the ResNet-50 classification results of a radiograph of a cat showing cardiomegaly, an alveolar pattern both in the cranial and caudal lungs, and pleural effusion. The activations of the last layer are visualized superimposed on the radiographs. Each image corresponds to the activations for a specific radiographic finding. The alveolar pattern **(B)** and the pleural effusion **(F)** were correctly identified by the model. However, the model failed to identify the cardiomegaly **(G)**. **(A)** Original image, **(B)** alveolar pattern, **(C)** unremarkable, **(D)** pneumothorax, **(E)** mass, **(F)** pleural effusion **(G)** cardiomegaly, **(H)** bronchial pattern.

The complete classification results in the test set for ResNet 50 and for IncV3 are reported in Tables 2, 3, respectively. The results of the De Long test showed no significant differences in the performances of either architecture for all the included radiographic findings. The overall accuracy in the test set was 81.8% for InceptionV3 and 84.1% for ResNet50. A visual representation of the analysis results is provided in Figure 2.

**Table 2:** Performance of InceptionV3 in the test set.

**Radiographic finding**	**AUC**	**Sensitivity**	**Specificity**	**PLR**	**NLR**
Alveolar pattern	0.93 (0.86–1)	82.4 (56.6–96.2)	93.7 (85.8–97.9)	13 (5.4–31.2)	0.2 (0.1–0.5)
Bronchial pattern	0.88 (0.70–1)	81.8 (48.4–97.7)	83.5 (73.9–90.7)	5 (2.9–8.6)	0.2 (0.1–0.8)
Cardiomegaly	0.71 (0.53–0.88)	50 (21.1–78.9)	83.3 (73.6–90.6)	3 (1.4–6.3)	0.6 (0.3–1)
Mass	0.54 (0.22–0.853)	33.3 (4.33–77.7)	86.7 (77.9–92.9)	2.5 (0.7–8.7)	0.8 (0.4–1.4)
No finding	0.86 (0.79–0.94)	83.3 (70.7–92.1)	76.2 (60.6–88)	3.5 (2–6.1)	0.2 (0.1–0.4)
Pleural effusion	0.99 (0.97–1)	85.7 (42.1–99.6)	94.4 (87.4–98.2)	15.3 (6.2–37.7)	0.2 (0–0.9)
Pneumothorax	0.78 (0.52–1)	66.7 (22.3–95.7)	78.9 (69–86.8)	3.2 (1.6–6.3)	0.4 (0.1–1.3)

**Table 3:** Performance of ResNet50 in the test set.

**Radiographic finding**	**AUC**	**Sensitivity**	**Specificity**	**PLR**	**NLR**
Alveolar pattern	0.95 (0.9–1)	82.5 (56.6–96.2)	93.7 (85.8–97.9)	13 (5.4–31.3)	0.2 (0.1–0.5)
Bronchial pattern	0.94 (0.84–1)	81.8 (48.22–97.7)	94.1 (86.8–98)	13.9 (5.7–34)	0.2 (0.1–0.7)
Cardiomegaly	0.71 (0.55–0.85)	66.7 (34.9–90)	67.9 (56.8–77.6)	2 (1.2–3.4)	0.5 (0.2–1)
Mass	0.58 (0.24–0.92)	33.3 (4–77.7)	81.1 (71.5–88.6)	1.7 (0.5–5.9)	0.8 (0.4–1.5)
No finding	0.86 (0.79–0.94)	92.6 (82.1–97.9)	66.7 (55.5–80.4)	2.8 (1.8–4.3)	0.11 (0 – 0.3)
Pleural effusion	0.97 (0.95–1)	85.7 (42.1–99.7)	96.6 (90.5–99.3)	25.4 (8–80.5)	0.2 (0–0.91)
Pneumothorax	0.83 (0.7–0.96)	33.3 (4.3–77.7)	90 (81.9–95.3)	3.3 (0.9–12)	0.74 (0.4–1.3)

## Discussion

An AI-based algorithm for the automatic detection of some of the most common radiographic findings in cats was developed. The high classification accuracy on the test set for some of the included radiographic findings, in particular alveolar pattern, bronchial pattern, no findings, pleural effusion, and pneumothorax, suggests that the developed CAD algorithm could potentially be used to assist veterinarians in interpreting feline thoracic radiographs. To fully investigate the usefulness of the proposed CNN, the error rate of the veterinarians in the detection of the above radiographic findings should also be investigated. Interestingly, the accuracy of this CAD in detecting the above-mentioned radiographic findings was comparable to the results reported for dogs (9, 10) and humans (16, 17), even though the database used for training was significantly smaller. A possible explanation is that the greater homogeneity in terms of body size and shape of cats might have reduced the intrinsic variability in the database thus enabling the CAD to achieve a high accuracy on the test set despite the reduced size of the database.

The accuracy for mass detection was low for both the tested CNN architectures. Interestingly, also in dogs (9, 10) the accuracy of CNNs in the detection of masses is lower than for the other radiographic findings. Instead, the reported accuracy for such a radiographic finding is reported to be high in human studies (16). It is the authors' opinion that, such a difference is, most likely, due to the presence of several mass-like structures (e.g., nipples, degeneration of costochondral joints) in normal canine and feline thoracic radiographs. Another possible explanation is that such a low accuracy might be related to the combination of the variable dimensions and locations of the masses within the thorax and the limited size of the training database.

Accuracy in detection of cardiomegaly was also lower than for the other radiographic findings. The radiographic identification of cardiomegaly in cats is challenging and, although some guidelines are currently available (13), its interpretation is often very subjective, especially in mild cases. Left atrial enlargement (the so called “valentine” heart) is a common finding in cats with cardiac disease (13) and is often better detected in dorsoventral rather than lateral projection. The low accuracy in detecting cardiomegaly evident in this study might be related to the fact that the CNN was trained only on lateral images and that the information on dorsoventral projections was unavailable during training. More in general, current guidelines on the classification, diagnosis and management of cardiomyopathies in cats (18) state that radiology is an insensitive method for detecting cardiac disease in cats and that cats with congestive heart failure may present radiologically normal cardiac silhouettes. A possible way to overcome such a limitation could be to train a CNN on feline thoracic radiographs classified based on the results of echocardiographic examinations, and then to test whether this CNN provides more accurate results than an experienced operator.

Recent studies (19) highlighted that, when trained on databases from different institutions, the generalization performances of CNNs depend on the disease prevalence in each database. Furthermore, the above-mentioned study also highlighted that CNNs trained on pooled data from different sites performed better on the data from these sites but not on external data. In the present study, the database used to train the network contained pooled data from two different institutions using three different X-ray equipments. Due to the limited size of the available database, the CAD performance differences regarding the data from each individual institution were not tested. However, training the models on pooled data from different institutions is reported as providing better generalization performances than training the model on data generated from a single institution (19).

The two CNN architectures tested in this study, ResNet 50 and IncV3, have been widely used both in human (20) and in veterinary medicine (21, 22) for the classification of diagnostic images. Both architectures have been engineered for the classification of everyday images and do not contain any radiology specific features. Furthermore, to improve performance, both CNNs were pre-trained on a large-scale database of everyday images, called ImageNet (www.image-net.org), and then fine-tuned on the feline database. It is the authors' opinion that the high classification accuracy achieved in the test set for several of the included radiographic findings might be, at least partially, due to the high standardization of the radiographic images. In fact, everyday images are often messy, and the same subject might come in different sizes; different shapes and colors might be in the foreground or background and so on. Instead, radiographs are acquired by skilled personnel in a highly standardized fashion. Interestingly, no statistically significant differences were evident in the performances of the two CNN architectures for any of the included radiographic findings.

A limitation of this study is that, due to the small database size, the number of radiographic findings included to train the CAD algorithm is smaller compared to those included in canine (9–12) and human studies (16). It is the authors' opinion that, at this stage of development, the proposed CAD could be more useful during emergency assistance, where the prompt identification of some of the included radiographic findings, in particular alveolar pattern, pleural effusion, and pneumothorax, is very important. The main advantage of using CNNs to develop CADs is that they are relatively easy to implement. Indeed, once the parsing modes have been defined, the individual radiographic findings can be directly selected or excluded for training.

To improve classification performances only correctly positioned and exposed radiographs were included in the database used for training. Therefore, the performance of the developed algorithm might be slightly different when used on technically incorrect images. Another limitation is that cross validation was not used and, given the limited size of the available database, different results are to be expected if other random splits are used. On the other hand, cross validation is not commonly used when CADs for the automatic classification of thoracic radiographs are developed, even in case of small sized data bases for training (8, 12).

## Conclusions

A CAD algorithm for the automatic detection of some radiographic findings in feline thoracic radiographs is proposed. This CAD showed a high accuracy in the identification of alveolar pattern, bronchial pattern, no findings, pleural effusion, and pneumothorax. The accuracy in identifying cardiomegaly was moderate whereas the accuracy in the identification of masses was low. The use of a larger database for training could, potentially, provide more accurate results. The developed CAD can be easily upgraded by simply adding new images to the database used for training, validation, and testing. Further testing on images acquired with different of X-Ray equipment will provide more insights in the performances of the developed CAD.

## Data Availability Statement

The data sets generated during and analyzed during the current study are not publicly available because they are property of the Veterinary Teaching Hospital of the University of Padua but are available from the corresponding author on reasonable request.

## Ethics Statement

This study was conducted respecting the Italian law 26/2014 (that transposes the EU directive 2010/63/EU). As the data used in this study were part of routine clinical activity, no Ethical Committee approval was needed. Informed consent regarding the treatment of personal data was obtained from the owners.

## Author Contributions

TB conceived the study, evaluated the radiographs, and drafted the manuscript. MW and HM developed the CNNs and drafted the manuscript. AZ, FT, CD, and FS evaluated the radiographs and drafted the manuscript. All authors contributed to the article and approved the submitted version.

## Funding

The present paper is part of a project funded by a research grant from the Department of Animal Medicine, Production and Health–MAPS, University of Padua, Italy: SID–Banzato 2019 (€ 15674; Development of an algorithm for the automatic classification and identification of the lesions on the radiographs of the thorax in dogs).

## Conflict of Interest

The authors declare that the research was conducted in the absence of any commercial or financial relationships that could be construed as a potential conflict of interest.

## Publisher's Note

All claims expressed in this article are solely those of the authors and do not necessarily represent those of their affiliated organizations, or those of the publisher, the editors and the reviewers. Any product that may be evaluated in this article, or claim that may be made by its manufacturer, is not guaranteed or endorsed by the publisher.

## References

[1] Bruno MA Walker EA Abujudeh HH. Understanding and confronting our mistakes: the epidemiology of error in radiology and strategies for error reduction. RadioGraphics. (2015) 35:1668–76. 10.1148/rg.2015150023 26466178

[2] Waite S Farooq Z Grigorian A Sistrom C Kolla S Mancuso A. A review of perceptual expertise in radiology-how it develops, how we can test it, and why humans still matter in the era of artificial intelligence. Acad Radiol. (2020) 27:26–38. 10.1016/j.acra.2019.08.018 31818384

[3] Waite S Kolla S Reede D Gale B Fuchs T Scott J. Interpretive error in radiology. Am J Roentgenol. (2016) 208:739–49. 10.2214/ajr.16.16963 28026210

[4] Nam JG Park S Hwang EJ Lee JH Jin KN Lim KY. Development and validation of deep learning-based automatic detection algorithm for malignant pulmonary nodules on chest radiographs. Radiology. (2019) 290:218–28. 10.1148/radiol.2018180237 30251934

[5] Sim Y Chung MJ Kotter E Yune S Kim M Do S. Deep convolutional neural network–based software improves radiologist detection of malignant lung nodules on chest radiographs. Radiology. (2020) 294:199–209. 10.1148/radiol.2019182465 31714194

[6] Lakhani P Prater AB Hutson RK Andriole KP Dreyer KJ Morey J. Machine learning in radiology: applications beyond image interpretation. J Am Coll Radiol. (2017) 15:350–9. 10.1016/j.jacr.2017.09.044 29158061

[7] Lee SM Seo JB Yun J Cho YH Vogel-Claussen J Schiebler ML. Deep learning applications in chest radiography and computed tomography. J Thorac Imaging. (2019) 34:75–85. 10.1097/RTI.0000000000000387 30802231

[8] Lakhani P Sundaram B. Deep learning at chest radiography: automated classification of pulmonary tuberculosis by using convolutional neural networks. Radiology. (2017) 284:574–82. 10.1148/radiol.2017162326 28436741

[9] Banzato T Wodzinski M Burti S Osti VL Rossoni V Atzori M. Automatic classification of canine thoracic radiographs using deep learning. Sci Rep. (2021) 11:1–8. 10.1038/s41598-021-83515-3 33597566 PMC7889925

[10] Boissady E de La Comble A Zhu X Hespel AM. Artificial intelligence evaluating primary thoracic lesions has an overall lower error rate compared to veterinarians or veterinarians in conjunction with the artificial intelligence. Vet Radiol Ultrasound. (2020) 61:619–27. 10.1111/vru.12912 32996208

[11] Burti S Osti VL Zotti A Banzato T. Use of deep learning to detect cardiomegaly on thoracic radiographs in dogs. Vet J. (2020) 262:105505. 10.1016/j.tvjl.2020.105505 32792095

[12] Li S Wang Z Visser LC Wisner ER Cheng H. Pilot study: application of artificial intelligence for detecting left atrial enlargement on canine thoracic radiographs. Vet Radiol Ultrasound. (2020) 61:611–8. 10.1111/vru.12901 32783354 PMC7689842

[13] Guglielmini C Diana A. Thoracic radiography in the cat: Identification of cardiomegaly and congestive heart failure. J Vet Cardiol. (2015) 17:S87–S101. 10.1016/j.jvc.2015.03.005 26776597

[14] He K Zhang X Ren S Sun J. Deep residual learning for image recognition. In: 016 IEEE Conference on Computer Vision and Pattern Recognition. Las Vegas, NV: IEEE (2016). p. 770–8. 10.1109/CVPR.2016.90

[15] Szegedy C Vanhoucke V Ioffe S Shlens J Wojna Z. (2015). Rethinking the Inception Architecture for Computer Vision. 10.1109/CVPR.2016.308

[16] Aggarwal R Sounderajah V Martin G Ting DSW Karthikesalingam A King D. Diagnostic accuracy of deep learning in medical imaging: a systematic review and meta-analysis. NPJ Digit Med. (2021) 4:65. 10.1038/s41746-021-00438-z 33828217 PMC8027892

[17] Guan Q Huang Y. Multi-label chest X-ray image classification via category-wise residual attention learning. Pattern Recognit Lett. (2020) 130:259–66. 10.1016/j.patrec.2018.10.027

[18] Luis Fuentes V Abbott J Chetboul V Côté E Fox PR Häggström J. ACVIM consensus statement guidelines for the classification, diagnosis, and management of cardiomyopathies in cats. J Vet Intern Med. (2020) 34:1062–77. 10.1111/jvim.15745 32243654 PMC7255676

[19] Zech JR Badgeley MA Liu M Costa AB Titano JJ Oermann EK. Variable generalization performance of a deep learning model to detect pneumonia in chest radiographs: a cross-sectional study. PLoS Med. (2018) 15:1–17. 10.1371/journal.pmed.1002683 30399157 PMC6219764

[20] Banzato T Causin F Puppa AD Cester G Mazzai L Zotti A. Accuracy of deep learning to differentiate the histopathological grading of meningiomas on MR images: a preliminary study. J Magn Reson Imaging. (2019) 50:1152–9. 10.1002/jmri.26723 30896065 PMC6767062

[21] Banzato T Bonsembiante F Aresu L Gelain ME Burti S Zotti A. Use of transfer learning to detect diffuse degenerative hepatic diseases from ultrasound images in dogs: a methodological study. Vet J. (2018) 233:35–40. 10.1016/j.tvjl.2017.12.026 29486877

[22] Banzato T Cherubini GB Atzori M Zotti A. Development of a deep convolutional neural network to predict grading of canine meningiomas from magnetic resonance images. Vet J. (2018) 235:90–2. 10.1016/j.tvjl.2018.04.001 29704946

